# Molecular pathways and therapeutic strategies in dermatofibrosarcoma protuberans (DFSP): unravelling the tumor’s genetic landscape

**DOI:** 10.17179/excli2024-7164

**Published:** 2024-05-14

**Authors:** Harpreet Singh, Heena Bholaram Choudhary, Deepa Satish Mandlik, Manoj Subhash Magre, Sourav Mohanto, Mohammed Gulzar Ahmed, Bhuvnesh Kumar Singh, Arun Kumar Mishra, Arvind Kumar, Amrita Mishra, T. Venkatachalam, Hitesh Chopra

**Affiliations:** 1 School of Pharmaceutical Sciences, IFTM University, Moradabad, Uttar Pradesh, 244102, India; 2 Department of Pharmacology, BVDU, Poona College of Pharmacy, Pune, 411038, Maharashtra, India; 3 Department of Pharmaceutics, Yenepoya Pharmacy College & Research Centre, Yenepoya (Deemed to be University), Mangalore, Karnataka, 575018, India; 4 Faculty of Pharmacy, Moradabad Educational Trust, Moradabad, Uttar Pradesh, 244001, India; 5 SOS School of Pharmacy, IFTM University, Moradabad, Uttar Pradesh, 244102, India; 6 School of Pharmaceutical Sciences, Delhi Pharmaceutical Sciences and Research University, New Delhi, 110017, India; 7 Department of Pharmaceutical Chemistry, JKKMMRFs-Annai JKK Sampoorani Ammal College of Pharmacy, Komarapalayam, The Tamil Nadu Dr. MGR Medical University, Chennai, Tamil Nadu, 638183, India; 8 Department of Biosciences, Saveetha School of Engineering, Saveetha Institute of Medical and Technical Sciences, Chennai - 602105, Tamil Nadu, India

**Keywords:** Dermatofibrosarcoma Protuberans, DFSP, cancer, genome, immunotherapy

## Abstract

Dermatofibrosarcoma Protuberans (DFSP) is a rare soft tissue sarcoma distinguished by its infiltrative growth pattern and recurrence potential. Understanding the molecular characteristics of DFSP is essential for enhancing its diagnosis, prognosis, and treatment strategies. The paper provides an overview of DFSP, highlighting the significance of its molecular understanding. The gene expression profiling has uncovered unique molecular signatures in DFSP, highlighting its heterogeneity and potential therapeutic targets. The Platelet-Derived Growth Factor Receptors (PDGFRs) and Fibroblast Growth Factor Receptors (FGFRs) signaling pathways play essential roles in the progression and development of DFSP. The abnormal activation of these pathways presents opportunities for therapeutic interventions. Several emerging therapies, i.e., immunotherapies, immunomodulatory strategies, and immune checkpoint inhibitors, offer promising alternatives to surgical resection. In DFSP management, combination strategies, including rational combination therapies, aim to exploit the synergistic effects and overcome resistance. The article consisting future perspectives and challenges includes the discovery of prognostic and predictive biomarkers to improve risk stratification and treatment selection. Preclinical models, such as Patient-derived xenografts (PDX) and genetically engineered mouse models, help study the biology of DFSP and evaluate therapeutic interventions. The manuscript also covers small-molecule inhibitors, clinical trials, immune checkpoint inhibitors for DFSP treatment, combination therapies, rational therapies, and resistance mechanisms, which are unique and not broadly covered in recent pieces of literature.

See also the graphical abstract(Fig. 1).

## Introduction

Dermatofibrosarcoma Protuberans (DFSP) is a rare form of skin cancer originating from fibroblast connective tissues. It is characterized by tumors that develop in the deep layers of the skin, particularly in the dermis and grow gradually (Llombart et al., 2018[114]). DFSP is primarily embodied in individuals within the young to middle-aged adult population, although it has the potential to occur in individuals of any age group. Despite being categorized as a low-grade malignancy, DFSP can potentially invade the surrounding tissues and recur following surgical removal (Baranov et al., 2020[14]). The precise etiology of DFSP remains elusive; nevertheless, it is postulated that genetic aberrations play a role in its pathogenesis. In most instances, the occurrence is sporadic, implying no identifiable predisposing factors involved (Jaju et al., 2016[85]). However, several DFSP cases have been linked to a specific chromosomal translocation involving the Collagen Type I Alpha 1 (COL1A1) and Platelet-Derived Growth Factor Beta (PDGFB) genes (Bhavani et al., 2023[17]). Genetic modification leads to the proliferation of cells and the development of tumors as a consequence of the excessive production of growth factor receptors (Bhavani et al., 2023[17]). Clinically, DFSP presents as a skin mass that is firm, slow-growing, and without any noticeable symptoms (Zhou et al., 2020[218]). The tumor typically appears as a raised, reddish-brown, or purple patch with a rubbery or woody consistency and evolves more eloquently and protuberantly, assuming a nodular or dome-like appearance. DFSP tends to affect specific body areas, i.e., the trunk, limbs, head, and neck (Bogucki et al., 2012[21]). The diagnosis of DFSP involves a comprehensive assessment that includes clinical evaluation, imaging modalities, and histopathological examination. The standard procedure involves performing a skin biopsy to acquire a tissue specimen for subsequent microscopic analysis (Ashrafzadeh and Fedeles, 2023[6]). DFSP is histologically distinguished by the presence of spindle-shaped cells that exhibit a storiform pattern resembling the shape of a cartwheel (Sirvent et al., 2003[165]). Immunohistochemistry is often used to confirm a diagnosis of DFSP by checking for specific markers, i.e., CD34 and vimentin in DFSP cells (Fuertes et al., 2013[57]). The primary therapeutic approach for DFSP involves the surgical excision of the tumor. The standard procedure involves the complete removal of the tumor and a surrounding healthy tissue margin, intending to minimize the likelihood of tumor recurrence (Paradisi et al., 2008[140]). In addition to surgical resection, radiation therapy can be used as an adjunct or alternative treatment for DFSP when complete removal is unattainable. Chemotherapy is usually reserved for advanced or metastatic cases (Wilder et al., 2015[201]).

When emphasis is laid on geographical distribution of DFSP, it is said that in the age group of 20 to 50 years, DFSP may occur and its occurrence is almost nil in new born and aged persons of 80 years and above age. General assumption is associated with the fact that approximately 16 thousands new cases and approx. 6 thousands deaths will be associated with death in United States itself. An estimated 0.8 to 4.5 cases per million individuals per year are associated with DFSP (Kreicher et al. 2016[98]; Rouhani et al. 2008[150]). No obvious racial bias has been shown in prior investigations, and it has been documented in people of all ethnicities. It has been found that Black individuals are more likely to develop Bednar tumor, a rare pigmented type of DFSP (Simon et al. 1997[164]). 

In general, the prognosis for DFSP is favorable as it has a low metastatic potential. The primary challenge, however, is preventing local recurrence, which can occur in 10-20 % of cases (Brahmachari et al., 2021[24]). It is crucial to have regular follow-up appointments to detect any recurrence or spread of the disease. In certain instances, targeted therapeutic interventions that effectively suppress aberrant platelet-derived growth factor (PDGF) receptor function have exhibited encouraging outcomes in managing recurrent or untreatable DFSP (Pierotti et al., 2010[145]). Comprehending the molecular attributes of DFSP is of paramount importance for a multitude of reasons, encompassing the realms of diagnosis, prognosis, and therapeutic approaches. By elucidating the underlying molecular mechanisms and genetic alterations associated with DFSP, researchers and healthcare professionals can gain valuable insights into the disease's behavior and develop more targeted and effective treatments (Mohammed et al., 2021[130]). It has been observed that any change in epigenesis impacts on various physiological and pathological aspects of human body. Also the modification in genetics has gained significant importance in the area of skin cancer research especially DFSP. In case of DFSP, the fusion of COL1A1-PDGFB has been identified. For pathogenesis of DFSP, this fusion variant employs exons 25, 32 and 47 also. Methylation of histone on 27 lysine to form H3K27Me3 is done by this fusion and it is ultimately required for initial silencing of target genes. Apart from this, studies on transcriptome sequencing analysis have shown a strong upregulation of EZH-2 in DFSP which is related to epithelial mesenchymal transition. This suggests that EZH-2-mediated methylation may lead to transcriptional repression of p53 genes, which in turn favors the development of DFSP (Li et al. 2018[107]). 

Phosphoproteomics has been extensively used as a preclinical research tool to characterize the phosphorylated components of the cancer proteome. Advancement in the phosphoproteomics resulted into insights of new drug target, mode of action associated with disease progression, and lastly biomarker discovery. Studies on phosphoproteomics have investigated various signaling pathways and also examined the kinase network cycle, which was not covered by transcriptomic technologies. It is pertinent to mention that in studies related with expression related profiling 5520 genes at the mRNA level by employing microarrays has explored distinct molecular signatures for number of sarcoma including DFSP thus in turn providing new markers with importance of diagnosis (Noujaim et al. 2016[137]).

The precise diagnosis is crucial for understanding the molecular aspects of DFSP. Clinically, DFSP can mimic other skin lesions, making it challenging to distinguish it from benign conditions or other sarcomas (Trøstrup et al., 2022[179]). Nevertheless, highly precise molecular markers exist in the form of the specific chromosomal translocation t (17;22) (q22; q13), leading to the fusion of COL1A1 and PDGFB genes that can be found in cases of DFSP (Zhu et al., 2021[220]). Molecular techniques, such as FISH and PCR, can facilitate the identification of this genetic modification. These methods are crucial in verifying the diagnosis and differentiating DFSP from other neoplasms that share similar characteristics (Hao et al., 2020[70]). In addition, understanding the molecular characteristics of DFSP can shed light on its biological behavior and prognosis. Different genetic subtypes of DFSP, such as the classic variant with COL1A1-PDGFB fusion and rare variants with alternative fusion partners, may manifest with diverse clinical manifestations (Lee et al., 2022[103]). Identifying and characterizing specific fusion types can help healthcare professionals predict the course of the disease, personalize treatment, and provide appropriate surveillance strategies for patients with DFSP. These molecular subtypes are associated with a high risk of recurrence or metastasis, making their identification crucial for accurate treatment and follow-up care (Trøstrup et al., 2022[179]).

**Figure 2:**
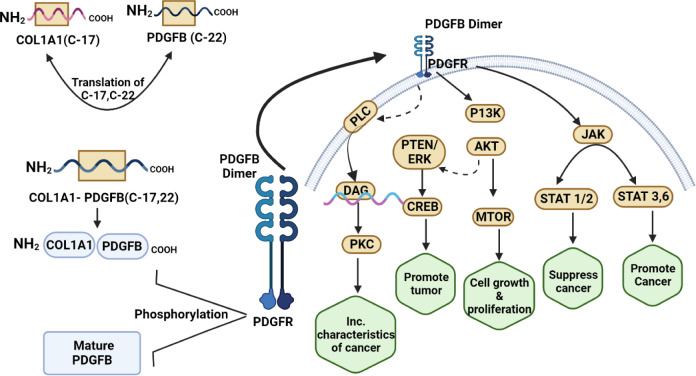
Mechanisms of tumor progression targeting PDGFR-B signaling pathway, contributed to the pathogenesis of DFSP (*created with Biorender.com*).

The molecular understanding of DFSP can also guide the development of targeted therapies. The fusion of COL1A1 and PDGFB causes overexpression of PDGF receptor beta (PDGFR-B), which plays a crucial role in the pathogenesis of DFSP (Tsagozis et al., 2020[180]). This understanding has led to the investigation of targeted therapies that specifically inhibit PDGFR-B signaling, depicted in Figure 2(Fig. 2). By inhibiting PDGFRs activity, the tyrosine kinase inhibitor imatinib has shown promising results in feasting advanced, recurrent, or unresectable DFSP cases (Koseła-Paterczyk and Rutkowski, 2017[96]). Other targeted therapies, i.e., nilotinib and sorafenib, have shown efficacy in a subset of patients with DFSP (Widmer et al., 2014[200]). In addition, molecular research on DFSP can contribute to ongoing efforts to elucidate the mechanisms underlying tumor progression, invasion, and metastasis (Liu et al., 2023[113]). By investigating the molecular alterations associated with DFSP, researchers can identify the critical pathways and signaling cascades that drive the growth and spread of tumors (Peng et al., 2022[143]). This paper explores the molecular aspects of DFSP, including genetic changes, chromosomal translocations, and signaling pathways that contribute to its development. Additionally, potential molecular targets for treating DFSP are discussed, including targeted and immunotherapeutic approaches. The study also examines small-molecule inhibitors, clinical trials, immune checkpoint inhibitors for DFSP treatment, combination therapies, rational therapies, and resistance mechanisms. The article highlights the challenges and prospects of DFSP research, such as identifying prognostic and predictive biomarkers, developing preclinical models, overcoming targeted therapy resistance, and improving personalized medicine for DFSP.

## Genomic Profiling of DFSP

### FISH

The Fluorescence in situ hybridization (FISH) is a genomic profiling technique that has proven extremely useful in studying DFSP. The utilization of FISH analysis plays a pivotal role in confirming the diagnosis of DFSP and in differentiating this particular tumor from other similar neoplasms (Chrzanowska et al., 2020[38]). FISH utilizes fluorescently labeled DNA probes that bind to specific target DNA sequences within tumor cells. FISH probes for DFSP were designed to target the COL1A1 and PDGFB genes or the breakpoints caused by their fusion. These probes were labeled with different fluorescent dyes, allowing specific genetic alterations to be visualized and identified under a fluorescence microscope (Schram et al., 2017[158]). In general, pathologists can detect the presence of the COL1A1-PDGFB fusion gene in DFSP tissue samples through FISH analysis (Segura et al., 2011[159]). The fusion gene was identified by juxtaposing fluorescent signals from the COL1A1 and PDGFB genes. The presence of the fusion gene, which is highly specific to DFSP, confirmed the diagnosis of DFSP (Segura et al., 2011[159]). The FISH analysis provided additional information regarding the genetic profile of DFSP. It can determine the frequency and distribution of the COL1A1-PDGFB fusion gene within tumor cells, which may differ between patients. This information can be used to assess tumor aggressiveness and predict the likelihood of recurrence (Karanian et al., 2015[91]). FISH analysis can also differentiate DFSP from other histologically similar tumors, such as benign fibrous histiocytoma or cellular dermatofibroma (Hornick, 2020[79]). Pathologists can utilize FISH to accurately diagnose DFSP tumors that do not have the COL1A1-PDGFB fusion gene, distinguishing them from other entities (Henry et al., 2023[74]).

### CMA

The Comprehensive genomic profiling (CMA) is a concise genomic profiling technique with numerous applications in DFSP research. It is essential to understand the genetic alterations and molecular characteristics of DFSP to develop targeted therapies and personalized treatment strategies (Dufresne et al., 2018[49]). By detecting copy number variations (CNVs) and loss of heterozygosity (LOH) across the entire genome, CMA enables researchers to gain valuable insights into the genomic landscape of DFSP (Peng et al., 2022[143]). In CMA, tumor tissue-extracted DNA is hybridized with microarrays containing thousands to millions of DNA probes (Levy and Wapner, 2018[106]). These probes are designed to target specific genomic regions. A patient's DNA sample was tagged with one fluorescent dye, whereas a reference DNA sample was tagged with another. CMA can identify regions of the genome exhibiting CNVs and LOH by comparing the fluorescence signals from the patient's DNA to the reference DNA (Shao et al., 2021[162]). 

The identification of CNVs and LOH in DFSP using CMA provides essential information about the genomic imbalances (Sharma et al., 2021[163]). The amplifications or deletions of distinct genes or genomic regions may indicate the involvement of critical genes in the pathogenesis of DFSP (Bridge, 2014[25]). For instance, identifying amplified genes may reveal potential oncogenes that drive the growth and progression of DFSP. In contrast, identifying deleted genes may reveal tumor suppressor genes that normally regulate cell division and growth (Bridge, 2014[25]). The identification of genomic alterations in DFSP can offer valuable insights into the disease's molecular mechanisms and aid the development of targeted therapies. CMA also contributes to the classification of DFSP subtypes at a molecular level. In most cases, DFSP is characterized by specific gene fusion events involving the COL1A1-PDGFB fusion gene (Saab et al., 2017[153]). However, only a small percentage of DFSP cases lack this fusion gene. CMA can assist in identifying alternative gene fusions or genomic rearrangements that may be present in these instances. The identification of these transformations can contribute to the enhancement of the molecular categorization of DFSP subtypes, potentially impacting prognosis and treatment alternatives (Linn et al., 2003[112]).

### PCR

The Polymerase Chain Reaction (PCR) is a fundamental technique in molecular biology with numerous applications in DFSP research. PCR permits the amplification of specific DNA sequences, enabling researchers to examine genetic alterations, mutations, and gene expression patterns related to DFSP (Tan et al., 2007[174]; Buteau al., 2018[28]). A series of heating and cooling cycles facilitated the denaturation of the DNA template, primer annealing, and DNA synthesis by a DNA polymerase enzyme during PCR. A small segment of DNA, known as the target sequence, is selectively amplified using primers that flank the target region. Typically, the PCR mixture consists of a DNA template, DNA polymerase enzyme, nucleotides, and primers (Lorenz, 2012[117]). Multiple applications of PCR have been reported in DFSP research. The identification and characterization of specific gene mutations or genetic alterations associated with the disease are distinct applications (Jain et al., 2010[84]). By targeting the mutated or altered regions of interest with designed primers, PCR can amplify and detect variations. PDGFB mutations are identified in DFSP genes, and through PCR, mutations in patient samples can be detected and analyzed (Hong et al., 2013[78]).

In addition, PCR can detect gene fusion events in DFSP. The COL1A1-PDGFB fusion gene is a defining characteristic of DFSP, and PCR-based techniques, such as reverse transcription PCR or multiplex PCR, can be used to detect the fusion transcript (Bridge and Cushman-Vokoun, 2011[26]). PCR can amplify and identify the presence of the fusion gene using primers that target the fusion junction, which diagnoses and classifies DFSP subtypes (Cerrone et al., 2014[32]). PCR can also be employed to investigate gene expression patterns in DFSP (Takahira et al., 2007[172]). The utilization of reverse transcription polymerase chain reaction (RT-PCR) enables the conversion of RNA molecules into their complementary DNA counterparts, facilitating subsequent amplification and quantification processes (Garibyan and Avashia, 2013[59]). Thus, this approach promotes the analysis of gene expression levels for specific genes of interest, including oncogenes, tumor suppressor genes, and other genes linked to DFSP. PCR-based gene expression analysis can offer valuable insights into the molecular mechanisms that underlie DFSP by comparing the expression levels of genes in normal and tumor tissues (Wu et al., 2019[204]).

### RNA-seq 

The RNA-seq (RNA sequencing) is a transformative genomic profiling technique with significant quantitative analysis in the study of DFSP (Gounder et al., 2022[66]). RNA-seq permits comprehensive analysis of the entire transcriptome, revealing gene expression levels, alternative splicing events, and gene fusion transcripts associated with DFSP (Xu et al., 2018[207]). The extraction of RNA molecules from DFSP tumor tissues is the first step in RNA-seq (Hofvander et al., 2019[77]). These RNA molecules encapsulate all cell transcripts, including protein-coding and non-coding RNAs. The extracted RNA was reverse-transcribed into complementary DNA (cDNA), which was then sequenced using high-throughput sequencing platforms (Talkish et al., 2014[173]).

RNA-Seq has numerous applications in DFSP research. RNA-seq allows for identifying differentially expressed genes in DFSP tumor tissues versus normal tissue samples (Hofvander et al., 2020[76]). Researchers can identify genes upregulated or downregulated in DFSP by comparing the abundance of transcripts, providing insights into the dysregulated molecular pathways and processes. This information can facilitate the identification of therapeutic targets and enhance our comprehension of the underlying biological mechanisms of DFSP (Laginestra et al., 2017[100]). Furthermore, RNA-seq enables the detection of alternative splicing events in DFSP (Ho et al., 2020[75]). Alternative splicing is a process by which various exon combinations within a gene are included or excluded, resulting in the production of multiple mRNA isoforms from a single gene (McAlinden et al., 2005[124]). RNA-seq analysis provides a comprehensive picture of DFSP alternative splicing patterns, identifying specific isoforms that may have functional implications for the disease. These isoforms can help DFSP development and progression by changing the protein structure, function, or expression levels (Rajagopal et al., 2022[146]). RNA-seq can also detect gene fusion transcripts, essential aspects of DFSP. DFSP is distinguished by the COL1A1-PDGFB fusion gene, which results from chromosomal translocation (Abbott et al., 2006[1]; Olson et al., 2018[138]). RNA-seq helps to confirm the presence of DFSP and contributes to the molecular classification of DFSP subtypes by detecting and characterizing fusion transcripts (Olson et al., 2018[138]).

### NGS & WES

The Next generation sequencing (NGS) is a cutting-edge genomic profiling technology that has revolutionized research on DFSP. NGS enables a thorough examination of the complete genome or specific regions of interest, providing valuable insights into the genetic modifications and molecular attributes associated with this ailment (Jin et al., 2021[88]). Whole-genome sequencing (WGS), whole-exome sequencing (WES), and targeted gene panel sequencing (TGPS) represent prominent examples of NGS methodologies (Han and Lee, 2020[69]). WGS entails sequencing the entire genome, providing a comprehensive view of the DFSP genetic landscape (Nguyen et al., 2023[134]). This method helps to identify various types of genetic changes, including single-nucleotide variants (SNVs), insertions and deletions (indels), CNVs, and structural variants (Nguyen et al., 2023[134]). WGS has the potential to reveal new mutations and changes in the DNA structure, leading to a better understanding of the genetic processes involved in DFSP (Wang et al., 2022[196]).

**Figure 3:**
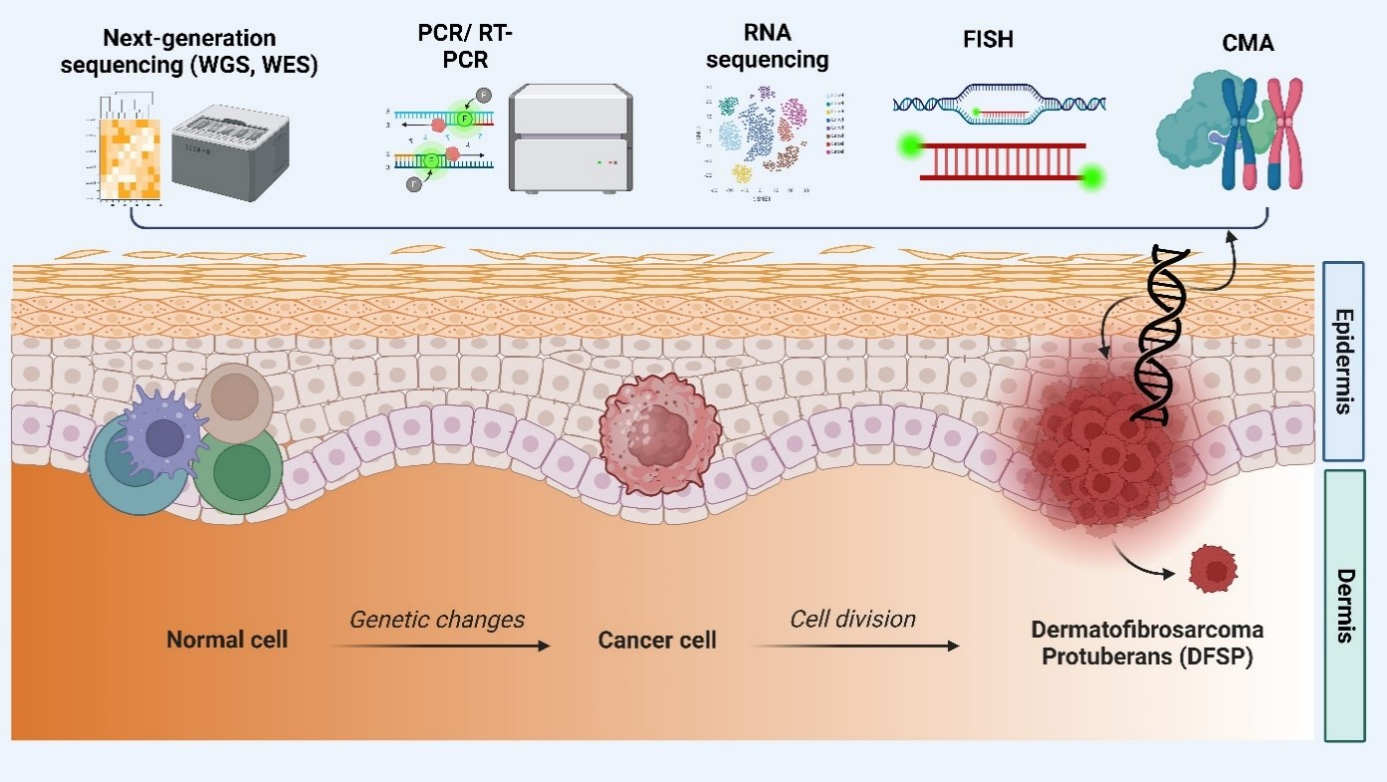
Schematic representation of different technologies for detecting DFSP by genomic profiling (*created with Biorender.com*).

In addition, WES primarily emphasizes the sequencing of the exome, which refers to the protein-coding regions of the genome (Suwinski et al., 2019[171]). Despite accounting for only a small portion of the genome, the exome contains a vast majority of disease-causing variants (Vinuesa et al., 2023[187]). WES allows the identification of SNVs and indels in DFSP protein-coding genes by sequencing the exome. This method is beneficial for detecting mutations in well-known cancer-related genes and pathways, shedding light on the genetic changes that drive DFSP development and progression (Cortes-Ciriano et al., 2023[39]). TGPS refers to the systematic identification and arrangement of a distinct group of genes that are recognized to be linked to DFSP or involved in pathways associated with cancer (Gounder et al., 2022[66]). This method enables focused analysis of relevant genes, providing in-depth coverage and detecting low-frequency mutations and fusion transcripts. Targeted gene panel sequencing is a cost-effective and efficient strategy (Gounder et al., 2022[66]). Figure 3(Fig. 3) depicts various genomic profiling methodologies employed in the analysis of DFSP.

NGS can help identify DFSP-specific driver mutations, oncogenic pathways, and therapeutic targets in research (Cassinelli et al., 2016[31]). NGS can also help with the molecular classification of DFSP subtypes by detecting fusion genes, such as the COL1A1-PDGFB fusion gene, which is common in DFSP (Wei et al., 2022[198]). Furthermore, NGS can reveal patterns of genomic alterations that may be diagnostic or prognostic, thereby assisting in personalized treatment approaches (Cassinelli et al., 2016[31]). NGS data analysis in DFSP research requires sophisticated bioinformatics pipelines to identify and interpret genetic alterations accurately. Integrating NGS data with other genomic and clinical data can help provide a more complete understanding of DFSP biology and aid in developing targeted therapies (Wei et al., 2022[198]). Table 1(Tab. 1) (References in Table 1: Asif et al., 2018[7]; Joshi and Deshpande, 2010[89]; Kukurba and Montgomery, 2015[99]; Serratì et al., 2016[160]; Vickers and Gibson, 2019[186]) comprehensively summarizes the advantages, limitations, and applications of various genomic profiling techniques in the context of DFSP.

## Understanding on Gene Expression and Signaling Pathways Associated in DFSP

Gene expression profiling in DFSP has emerged as a valuable tool for identifying potential therapeutic targets and understanding the molecular characteristics of the disease (Merry et al., 2021[127]). Gene expression profiling entails the concurrent assessment of the activity of numerous genes, thereby offering a comprehensive perspective on the genes that are either activated or inactive in DFSP tumors (Pennacchioli et al., 2012[144]). The main objective of gene expression profiling in DFSP is to gain a comprehensive understanding of the biological mechanisms and pathways involved in the initiation and advancement of this disease (Beck et al., 2010[15]). By comparing gene expression profiles between DFSP tumors and normal skin or other types of sarcomas, researchers have successfully identified significantly dysregulated genes and pathways specific to DFSP (Lai et al., 2017[101]). The dysregulated genes often contribute to cellular growth, proliferation, angiogenesis, and extracellular matrix remodeling, all of which are vital processes involved in the development and advancement of tumors (Winkler et al., 2020[203]). Multiple studies have reported the differential expression of specific genes and pathways in DFSP. Genes involved in the PDGF signaling pathway, including PDGFRB, PDGFB, and PDGFRA, are typically upregulated in DFSP (McCarthy et al., 2010[125]). The observed result aligns with the existence of the COL1A1-PDGFB fusion gene in most cases of DFSP, leading to abnormal activation of PDGF signaling. In addition, gene expression profiling has helped classify DFSP into distinct molecular subtypes, shedding light on the disease's heterogeneity. Based on gene expression patterns, one study identified two distinct subtypes of DFSP: the fibrosarcomatous subtype and the typical subtype (Bahrami and Folpe, 2010[11]). The fibrosarcomatous subtype exhibited elevated levels of gene expression related to fibroblast activation, extracellular matrix remodeling, and cell migration. These findings are consistent with its more aggressive behavior and heightened susceptibility to metastasis (Ulisse et al., 2009[184]).

The gene expression profiling technique was additionally employed in identifying prospective therapeutic targets in the context of DFSP. Through the examination of gene expression profiles in DFSP tumors, researchers have successfully identified specific genes that exhibit unique expression patterns or are excessively expressed in DFSP. These findings indicate the potential of these genes to serve as viable targets for therapeutic interventions (Bertucci et al., 2013[16]). For example, the fibroblast growth factor receptor 1 (FGFR1) gene is highly expressed in DFSP tumors, indicating that FGFR1 inhibitors may effectively target DFSP tumors (Roskoski, 2018[148]). Furthermore, gene expression profiling has not only facilitated the identification of potential therapeutic targets but has also enabled the prediction of patient outcomes and the guidance of treatment decisions. By analyzing gene expression signatures, researchers have developed gene expression-based prognostic models that can predict the likelihood of recurrence, metastasis, or overall survival in DFSP patients. These models may aid clinicians in determining the most appropriate treatment strategies and monitoring protocols for specific patients (Beck et al., 2010[15]).

### PDGF receptor signaling pathway

#### Role of PDGFRs in DFSP development and progression

The PDGFRs are essential for the growth and progression of DFSP (Chen et al., 2021[34]). PDGFRs are cell surface proteins that bind to platelet-derived growth factors, which are specific growth factors (Wang et al., 2022[195]). These growth factors control cell division, proliferation, and migration and their abnormal activation has been linked to developing cancers such as DFSP (Yuan et al., 2021[213]). As previously mentioned, a notable characteristic of DFSP is a chromosomal translocation event leading to the fusion of two specific genes, namely COL1A1 and PDGFB. The fusion gene gives rise to a chimeric protein, which is accountable for the constitutive activation of PDGFRs (Köster et al., 2020[97]). Aberrant activation of the PDGFRs leads to unregulated cellular proliferation and division, thereby playing a significant role in the pathogenesis and advancement of DFSP (Timbergen et al., 2019[178]). Upon activation of the PDGFRs, a signaling cascade is initiated, subsequently activating multiple downstream pathways associated with cellular processes such as proliferation, survival, and migration (Li et al., 2021[109]). The Rat Sarcoma - Mitogen-Activated Protein Kinase (Ras-MAPK) pathway plays a crucial role in regulating cellular proliferation and differentiation, representing a significant signaling cascade activated by the PDGFRs. The persistent activation of this pathway, induced by the signaling of PDGFRs, facilitates the ongoing proliferation of DFSP cells, leading to the development of tumor nodules and skin protuberances (Rezatabar et al., 2019[147]). In addition, the activation of the PDGFRs triggers the phosphoinositide 3-kinase (PI3K)-Akt pathway, which is crucial in promoting cell survival and conferring resistance against apoptosis (Wang et al., 2022[197]). The activation of the pathway by the PDGFRs enhances the survival of DFSP cells and enables them to evade apoptosis, thereby facilitating tumor progression and conferring resistance to therapeutic interventions (Esteban-Villarrubia et al., 2020[52]).

#### Targeting PDGFRs as a therapeutic approach

**Figure 4:**
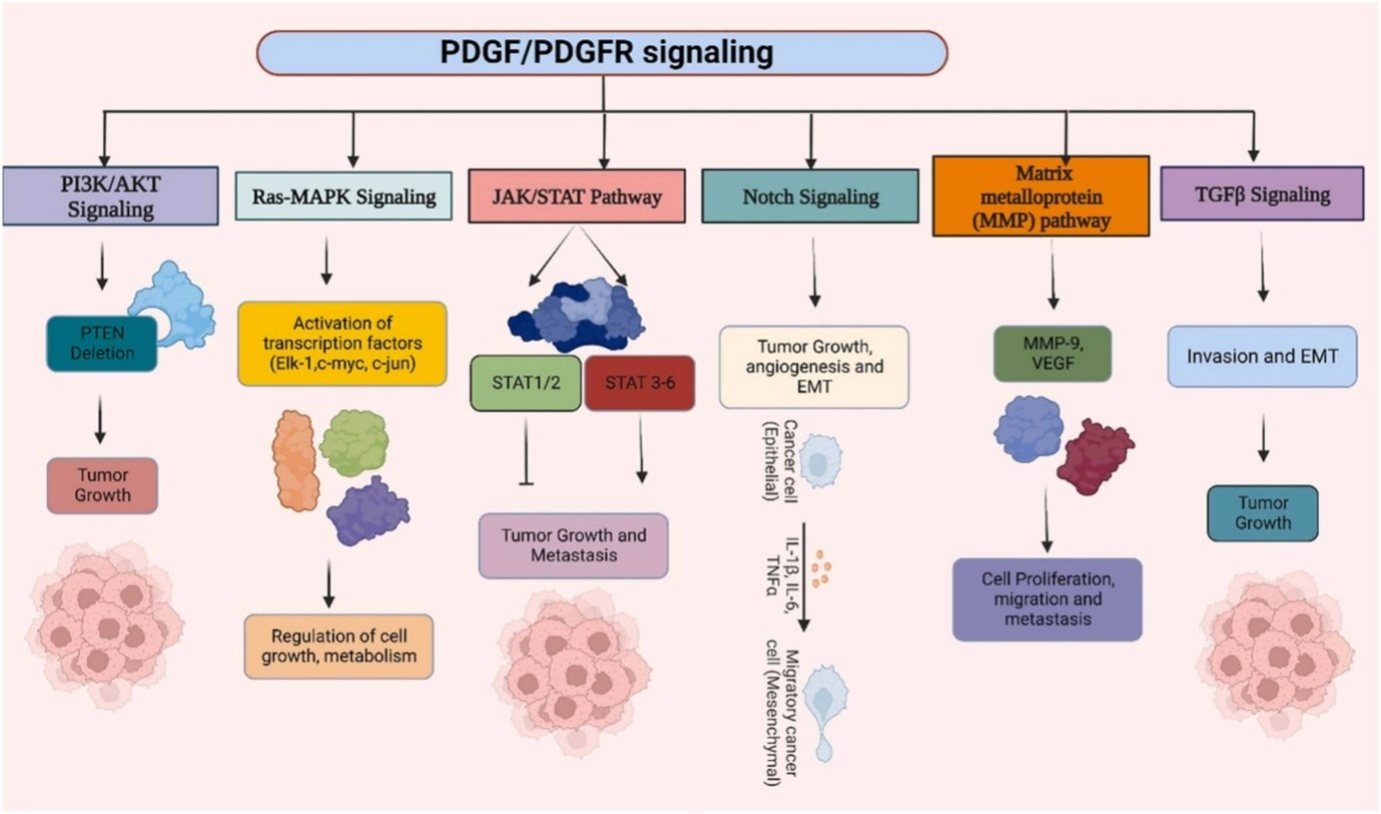
Understanding the cross-connection between PDGF/PGDFR signaling with other cellular signaling pathways in cancer progression, has further exhibited adequate consequences in DFSP treatment (Pandey et al., 2023).

Targeting the PDGFRs has emerged as a promising therapeutic strategy for treating DFSP (Zou et al., 2022[221]). DFSP is a rare form of skin cancer characterized by a chromosomal translocation that activates the PDGFRs constitutively (Ugurel et al., 2019[183]). The aberrant activation described is crucial for the advancement and growth of DFSP, thereby establishing the PDGFRs as a favorable target for therapeutic intervention. Inhibitors of small molecules specifically targeting the PDGFRs have demonstrated promise in treating DFSP (Yesilkanal et al., 2021[210]). Imatinib is one such inhibitor that has shown efficacy in inhibiting the abnormal PDGFRs signaling pathway (Zou et al., 2022[221]). The activation and downstream signaling of the PDGFRs are effectively suppressed by imatinib binding to the ATP-binding site (Tsioumpekou et al., 2020[181]). The specific inhibition of PDGFRs effectively suppresses cellular growth, proliferation, and viability, leading to the regression of tumors observed in individuals diagnosed with DFSP. According to clinical studies, the use of PDGFRs inhibitors in treating DFSP has yielded encouraging results (Pandey et al., 2023[139]) due to crosstalk between PDGFRs with other cellular pathways, shown in Figure 4(Fig. 4). Imatinib has been found to be effective in reducing tumor size, alleviating tumor-related symptoms, and increasing the overall survival rate of DFSP patients (Lee et al., 2023[104]). It has been especially useful in cases where surgical resection is difficult due to the tumor's location or its extensive involvement. Furthermore, the utilization of PDGFRs inhibitors has been observed as a potential approach for neoadjuvant therapy, aiming to diminish the size of tumors before surgical intervention and adjuvant therapy to prevent tumor recurrence subsequent to surgical removal (Nevola et al., 2023[133]).

However, it is essential to note that not all DFSP patients may react similarly to PDGFRs inhibitors. Individual factors, such as the specific molecular alterations in the tumor and the degree of PDGFRs activation, can influence the treatment response (Schneider et al., 2017[157]). Patients most likely to benefit from PDGFRs-targeted therapies can be identified through genetic testing and molecular profiling of DFSP tumors, allowing for a more personalized and effective treatment plan (Kondapalli et al., 2005[95]). In addition, the optimal treatment duration and long-term efficacy of PDGFRs inhibitors in DFSP are still under investigation (Rutkowski et al., 2017[151]). Over time, resistance to PDGFRs inhibitors can develop, resulting in tumor progression and recurrence (Weigel et al., 2013[199]). Various ongoing research focuses on understanding the resistance mechanisms and developing strategies to overcome them, such as combination therapies or developing next-generation inhibitors with enhanced potency and selectivity (Park et al., 2018[141]). Targeting the PDGFRs represents a promising therapeutic strategy for treating DFSP, as previously stated (Cuppens et al., 2017[41]). Small-molecule inhibitors, such as imatinib, have effectively inhibited the abnormal PDGFRs signaling pathway and induced tumor regression in patients with DFSP. To maximize the benefits of PDGFRs-targeted therapies in DFSP, however, additional research is required to optimize treatment approaches, identify predictive response markers, and overcome resistance mechanisms (Rössler et al., 2008[149]).

### FGFR signaling pathway

#### Involvement of FGFRs in DFSP pathogenesis

**Figure 5:**
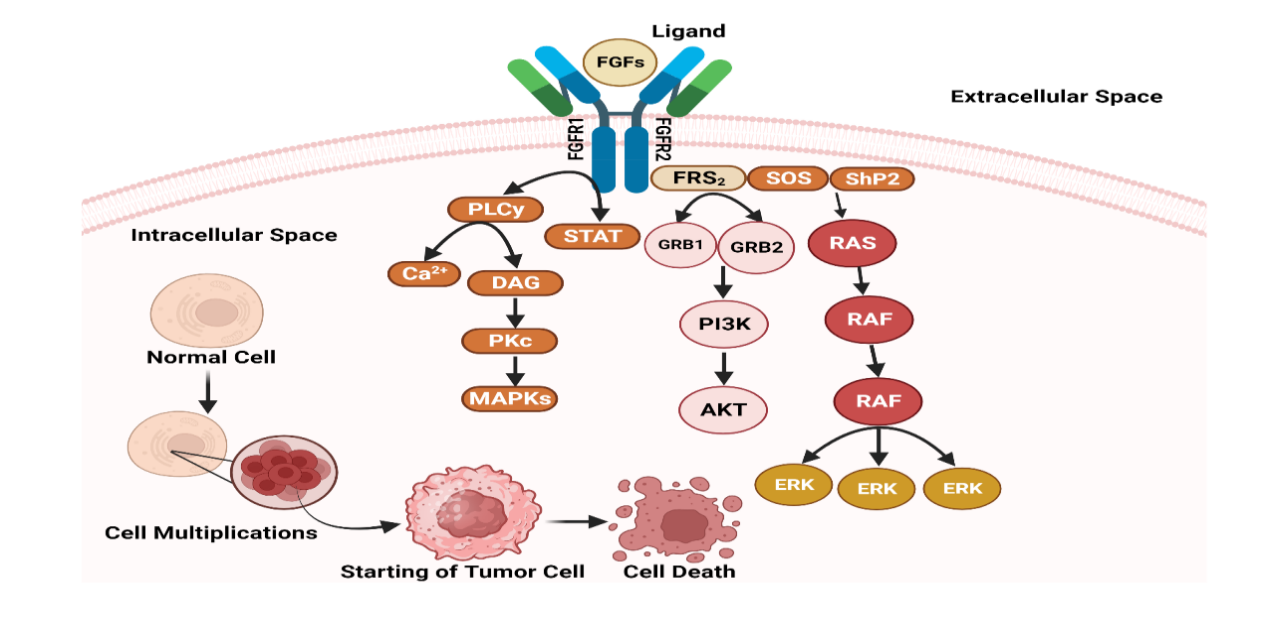
Understanding of FGFs/FGFR signaling pathway in pathogenesis of DFSP (*created with Biorender.com*)

The FGFRs are a family of receptor tyrosine kinases important for cell growth, differentiation, and tissue development (Gavine et al., 2012[60]). According to contemporary research, activating FGFRs signaling abnormally has been linked to various cancers, including DFSP (Heldin et al., 2018[73]). Autocrine or paracrine activation is one mechanism by which FGFRs contribute to DFSP pathogenesis (Cassinelli et al., 2016[31]). DFSP cells have been found to express FGFRs, specifically FGFR1 and FGFR2, as well as their ligands, fibroblast growth factors (FGFs) (Zhou et al., 2016[217]). When DFSP cells produce FGFs, autocrine activation occurs as the produced FGFs bind to FGFRs in the same cells. This results in continuous FGFR signaling, promoting cell proliferation, survival, and migration, ultimately leading to tumor growth (Burger, 2011[27]). The paracrine activation of FGFRs signaling in DFSP has been observed in addition to autocrine activation (Baird et al., 2005[12]). FGFs are produced by stromal cells, such as fibroblasts and endothelial cells, which can activate FGFRs on DFSP cells (Cao, 2013[29]). This paracrine activation enhances DFSP cells' malignant behavior by stimulating their growth and invasiveness. In addition, the fusion protein formed by the COL1A1-PDGFB translocation in DFSP has been shown to enhance FGFRs signaling (Esteban-Villarrubia et al., 2020[52]). This fusion protein has been shown to interact with FGFRs and increase their phosphorylation, resulting in increased downstream signaling cascades that promote tumor growth and survival. The signaling pathway of FGFs and FGFRs in the pathogenesis of DFSP is depicted in Figure 5(Fig. 5).

#### Potential targeting of FGFRs for therapeutic intervention in DFSP

The role of FGFRs signaling in the pathogenesis of DFSP has important implications for potential therapeutic strategies (Madhusudan and Ganesan, 2004[119]). Targeting FGFRs and their downstream signaling pathways could pave the way for developing novel DFSP therapies (Roskoski, 2018[148]). Several FGFR inhibitors are being studied in preclinical and clinical trials, demonstrating their potential as DFSP-targeted therapies (Demicco et al., 2012[47]; Sethi and Keedy, 2016[161]). The mechanism of action of small-molecule inhibitors targeting FGFRs involves their binding to the ATP-binding site of the receptor, thereby impeding the process of phosphorylation and subsequent activation of downstream signaling pathways (Sethi and Keedy, 2016[161]). In the context of DFSP models, the inhibitors mentioned above demonstrate a notable capacity to impede the signaling of FGFRs effectively. This consequential effect manifests as a reduction in cellular proliferation, apoptosis induction, and tumor growth inhibition (Alvarez et al., 2006[4]). The preclinical findings, which have shown promise, have established a foundation for conducting clinical trials to assess the safety and effectiveness of FGFR inhibitors in patients with DFSP (Wilding et al., 2019[202]).

**Figure 6:**
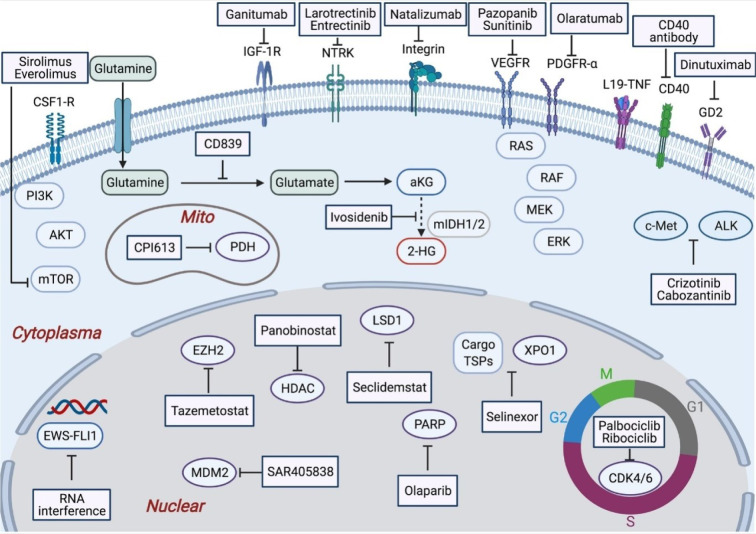
Depicted are various types of sarcomas (i.e., 100 subtypes), which are characterized by high heterogeneity in molecular profiles. However, certain sarcomas exhibit typical molecular and genetic features, which have led to the development of drugs approved for their treatment. Early targeted therapies for sarcomas have included anti-angiogenesis, which has shown favorable outcomes, particularly in highly vascularized tumors such as ASPS. Other translational targeted drugs have been developed based on signals from molecules such as PARP, IGF-1R, CDK4/6, mTOR, and c-MET, which are clinically evaluated for sarcomas with corresponding signals. Some previously untraceable targets, such as TP53 and fusion proteins, have also progressed. Notably, the EWS/FLI1 in Ewing sarcomas and the NTRK in infantile fibrosarcoma have shown encouraging developments. Additionally, promising strategies in sarcomas include epigenetic drugs and drugs targeting metabolism. Advancements in the biological exploration of sarcomas have also led to significant therapeutic potential in ADC drugs, anti-tumor antibodies, cytokine-antibody drugs, and drugs for CRM1, GPR20, and integrin (Tang et al., 2021).

Various clinical trials reported that using FGFR inhibitors in sarcoma has yielded promising results, further summarized in Figure 6(Fig. 6) (Reference in Figure 6: Tang et al., 2021[175]). These trials resulted at response rates, duration of response, and overall survival in patients who have been given FGFR-targeted therapies (Yashiro and Matsuoka, 2016[208]). Early-stage clinical trials have shown promising results, with significant tumor shrinkage and disease stabilization observed in DFSP patients treated with FGFR inhibitors (Yashiro and Matsuoka, 2016[208]). In some cases, complete responses have been reported, indicating that FGFR inhibitors can potentially induce tumor regression in DFSP (Mendel et al., 2003[126]). Targeted therapies offer a new treatment alternative for patient's ineligible for surgery or who have advanced or metastatic DFSP that cannot be effectively managed with other treatment methods (Iqbal and Iqbal, 2014[82]). However, it is important to note that the response to FGFR inhibitors varies between patients. DFSP tumor genetic profiling can aid in identifying specific alterations in FGFR genes, providing valuable information on the potential efficacy of FGFR-targeted therapies (Sborov and Chen, 2015[156]).

Furthermore, resistance mechanisms to FGFR inhibitors are a concern, emphasizing the importance of ongoing research to overcome therapeutic resistance and improve treatment strategies (Tang et al., 2021[175]). Clinical trials are also looking into FGFR inhibitor-based combination therapies (Mahipal et al., 2020[120]). These combinations aim to improve treatment efficacy by targeting multiple signaling pathways involved in DFSP pathogenesis at the same time (Aggarwal et al., 2009[2]). Synergistic effects may be achieved by combining FGFR inhibitors with other targeted therapies or conventional chemotherapy agents, leading to improved tumor regression and patient outcomes (Li et al., 2023[108]; Rutkowski et al., 2010[152]; Steeghs et al., 2007[170]).

## Emerging Therapeutic Strategies for DFSP Management

### Surgical resection

Surgical resection is the primary therapeutic approach for the management of DFSP. The primary objective of this surgical procedure is to achieve complete removal of the tumor while minimizing the likelihood of its reoccurrence and maintaining favorable functional and aesthetic results (Alshaygy et al., 2023[3]; Monnier et al., 2006[131]; Vukadinovic et al., 2007[189]). A thorough preoperative assessment is performed before surgical resection (Saiag et al., 2015[154]). In order to establish a definitive diagnosis, medical professionals employ various diagnostic methods, including a physical examination, imaging techniques such as ultrasound or MRI, and a biopsy (Saiag et al., 2015[154]). In order to ascertain the optimal surgical methodology, an evaluation is conducted to assess the magnitude of the tumor and its interrelation with adjacent anatomical components (Zheng et al., 2015[215]; Ge et al., 2009[61]). Once the surgical plan has been developed, the procedure is performed under general anesthesia to ensure patient comfort and safety (Yu et al., 2008[212]). The surgical procedure commences with the surgeon initiating an incision in the integument, guided by pertinent factors such as the precise anatomical site, dimensions, and extent of the neoplasm. The surgical incision is carefully strategized to ensure sufficient visibility and entry to the tumor (Yu et al., 2008[212]).The goal is to achieve clear margins, meaning the tumor is completely excised and surrounded by a rim of healthy tissue. The extent of resection may differ depending on the characteristics of the tumor and its proximity to critical structures (Veronese et al., 2017[185]). The surgeon may employ different techniques, such as end bloc resection or Mohs micrographic surgery, to achieve comprehensive tumor cell removal and mitigate the potential for residual cells (Madani et al., 2000[118]). The surgeon may send tissue samples from the tumor bed to the pathology laboratory for frozen section analysis at any time during the surgical resection (Cui et al., 2022[40]). This allows for intraoperative margin assessment. If the margins are positive (tumor cells at the edges), additional tissue removal may be required to achieve clear margins (Loghdey et al., 2014[115]). Through this iterative process, the tumor is guaranteed to be entirely removed.

After the tumor has been removed, the surgeon will begin reconstruction. The reconstruction technique used will be determined by the size and location of the defect as well as the needs of the individual patient (Snow et al., 2004[167]). Primary closure (bringing the surrounding tissue together), skin grafts, local flaps (moving nearby tissue to cover the defect), and even more complex procedures such as free tissue transfer may be options for reconstruction (Bonomi et al., 2018[22]). Patients will need close monitoring and follow-up care after surgery to ensure proper wound healing and detect any signs of recurrence (Thornton et al., 2005[177]).

### Immunomodulatory approaches for DFSP management

The immunomodulatory approaches have emerged as promising DFSP management strategies. While surgical resection is the primary treatment option, alternative therapies are required in cases where surgery is not possible, or the tumor has advanced or metastasized (Grimer et al., 2010[68]). Immunomodulation is the deliberate alteration of the immune system to enhance its ability to recognize and combat cancerous cells (Wagle et al., 2011[191]). This method seeks to activate and strengthen the body's natural defences to recognize and eliminate cancer cells more effectively (Dancsok et al., 2019[42]). Adoptive cell therapy is one of the immunomodulatory approaches being investigated in treating DFSP (Fujimura, 2022[58]). This method entails injecting immune cells, such as tumor-infiltrating lymphocytes (TILs), specifically selected and expanded in the lab to target cancer cells (Sousa et al., 2021[168]). Adoptive cell therapy seeks to boost the anti-tumor immune response by supplying many potent immune cells capable of recognizing and attacking DFSP cells (Kerrison et al., 2022[93]). While research is still in its early stages, adoptive cell therapy shows promise as a potential treatment option for DFSP. Immunomodulation can also be accomplished through the use of cytokines. Cytokines are signaling molecules that regulate the behavior of immune cells and can be used to modulate the immune response to DFSP (Tazzari et al., 2017[176]). Interferon-alpha and interleukin-2 are two cytokines studied in the treatment of DFSP. They have demonstrated some efficacy in controlling tumor growth and preventing recurrence, but their use may be restricted due to potential side effects (Chen et al., 2016[35]).

#### Immune checkpoint inhibitors in DFSP treatment

Although surgery is frequently employed as the primary therapeutic approach for DFSP), recent studies have demonstrated the potential efficacy of immune checkpoint inhibitors in managing advanced or metastatic cases of DFSP (Yuan et al., 2021[214]). Immune checkpoint inhibitors represent a pharmacological class of agents that elicit an enhanced immune response within the human body, thereby facilitating the identification and targeted elimination of malignant cells (Dufresne et al., 2018[49]). Pembrolizumab is one of the most extensively studied immune checkpoint inhibitors in DFSP treatment (Ascierto and Schadendorf, 2022[5]). It inhibits the interaction between the programmed death-1 (PD-1) receptor on immune cells and the programmed death ligand-1 (PD-L1) on cancer cells (Choi et al., 2020[37]). Typically, this interaction suppresses the immune response and enables cancer cells to evade detection. Pembrolizumab enhances the ability of an immune system to recognize and eliminate DFSP cells by inhibiting this interaction (Forsythe et al., 2022[55]). The results of clinical trials evaluating the use of pembrolizumab in advanced DFSP are encouraging. Pembrolizumab demonstrated a high objective response rate in a phase II trial, with a significant proportion of patients experiencing tumor shrinkage (Jimenez et al., 2020[87]). In addition, the responses were long-lasting, with several patients maintaining disease control for an extended period. Pembrolizumab shows promise as a potential treatment option for patients with advanced DFSP who are ineligible for surgery or other standard therapies (Blume-Peytavi et al., 2019[20]).

Nivolumab is an additional immune checkpoint inhibitor that has shown promise in the treatment of DFSP (Stătescu et al., 2023[169]). Nivolumab, like pembrolizumab, targets the PD-1 receptor and has demonstrated efficacy in treating various cancers. In a case report, a patient with metastatic DFSP who had previously received multiple treatments was administered nivolumab as salvage therapy. The patient experienced a substantial decrease in tumor size and disease stabilization, highlighting the potential benefit of nivolumab in advanced DFSP cases (Janssens et al., 2021[86]). Atezolizumab, a form of immune checkpoint inhibitor, has demonstrated potential in the therapeutic management of various types of cancer (Zhu et al., 2020[219]). Although there is limited research on using atezolizumab in the context of DFSP, ongoing investigations explore its potential application in this particular condition. Atezolizumab effectively hinders the interaction between the PD-L1 expressed in cancer cells and the PD-1 receptor in immune cells (De Leo et al., 2020[44]). Atezolizumab functions by inhibiting the interaction between cancer cells and the body's immune system, thereby facilitating the restoration and augmentation of the immune response against cancer cells (Zhong et al., 2022[216]).

Various preliminary evidence suggests that atezolizumab may be helpful in the treatment of DFSP (Blay et al., 2020[19]). In one case report, a patient with metastatic DFSP was given atezolizumab after failing other treatments. The patient responded partially to atezolizumab therapy, with tumor regression and an improved overall condition (Yen and Chen, 2018[209]). This demonstrates atezolizumab's potential as a viable treatment option for advanced DFSP cases where surgery may be impractical (Yen and Chen, 2018[209]). However, it is crucial to acknowledge that the utilization of atezolizumab or any immune checkpoint inhibitor in dermatofibrosarcoma protuberans (DFSP) is still in the experimental stage (Lopes-Brás et al., 2022[116]). Continuing investigations are underway to ascertain the optimal dosage, duration, and potential synergistic effects when combined with other therapeutic approaches (Dematteo et al., 2002[46]). In addition, it is important to note that the response to immune checkpoint inhibitors may vary among patients, indicating the necessity for further investigation in order to identify biomarkers or predictive factors that can aid in the selection of patients who are more likely to derive therapeutic benefits from these treatments (Havel et al., 2019[72]). Several clinical trials are being conducted better to understand the efficacy and safety of atezolizumab in DFSP, and participation in these trials may provide eligible patients with access to this novel therapy (Lalan et al., 2021[102]).

### Combinatorial approaches in DFSP

#### Rational combination therapies in DFSP

The treatment of DFSP has evolved, and rational combination therapies have emerged as a promising approach to improving outcomes for patients with this rare disease (Dangoor et al., 2016[43]). One of the most effective DFSP combination therapies combines surgery and radiation (Lembo et al., 2021[105]). Surgical resection is the primary treatment for DFSP. However, because DFSP is infiltrative, achieving clear surgical margins can be difficult (Wacker et al., 2004[190]). Radiation therapy is frequently used as an adjuvant treatment in such cases to reduce the risk of local recurrence (Fassnacht et al., 2006[53]). Clinicians can improve local control rates and overall survival in DFSP patients by combining surgery and radiation therapy, reducing the risk of tumor regrowth and disease progression (Kepka et al., 2005[92]).

Imatinib, a tyrosine kinase inhibitor, is another promising combination therapy for DFSP (Kondapalli et al., 2005[95]). Imatinib has shown impressive anti-DFSP activity by explicitly targeting the PDGFRs pathway, which is frequently activated in DFSP due to a chromosomal translocation. Before surgery, neoadjuvant imatinib therapy can shrink the tumor, making complete resection easier and reducing the required surgery (Gold and Dematteo, 2006[64]; Lorenz, 2012[117]). Adjuvant imatinib therapy following surgery has also reduced the risk of local recurrence and distant metastasis (Kondapalli et al., 2005[95]). This rational combination approach improves long-term disease control and patient outcomes significantly. In addition to surgery and imatinib, researchers are investigating the possibility of combining other targeted therapies with traditional treatment modalities in DFSP (Frankel et al., 2011[56]). For example, in many patients, the combination of imatinib and methotrexate, a chemotherapy agent, has shown promising results. Methotrexate can improve the response to imatinib therapy by increasing the cytotoxic effect on DFSP cells (George et al., 2006[62]). This approach may be especially useful in cases where imatinib alone is not producing the best results. It is imperative to acknowledge that the implementation of rational combination therapies in treating DFSP necessitates a personalized approach that considers various factors, including tumor size, location, and molecular characteristics. Moreover, further investigation and clinical trials are necessary to examine the effectiveness and safety of different combination strategies and identify biomarkers that can aid in predicting the response to particular treatments. Ultimately, integrating rational combination therapies exhibits significant potential in DFSP treatment. The integration of surgical procedures and radiation therapy, alongside the administration of targeted therapy imatinib, has demonstrated substantial advancements in local control rates, overall survival, and disease-free intervals (Wang et al., 2012[194]). The combination of imatinib with other agents, such as methotrexate, can potentially improve treatment outcomes even further. Continued research and collaboration between clinicians and researchers are critical for refining and expanding the repertoire of rational combination therapies for DFSP, ultimately improving the prognosis and quality of life for patients suffering from this rare disease (Atkinson and Gilbertson, 2011[8]). Furthermore, the advantages and disadvantages of various treatment approaches for DFSP are summarized in Table 2(Tab. 2) (References in Table 2: Badhey et al., 2021[10]; Tazzari et al., 2017[176]; Ugurel et al., 2019[183]; Vitiello et al., 2022[188]).

#### Synergistic effects and overcoming resistance

Synergistic effects and overcoming resistance are important DFSP management considerations (Imai and Takaoka, 2006[81]). Synergistic effects occur when two or more treatment modalities or drugs enhance each other's therapeutic efficacy and tumor control. The development of strategies to circumvent or combat the mechanisms that lead to treatment resistance in DFSP is required to overcome resistance (Bikker et al., 2009[18]). These factors are crucial for optimizing treatment outcomes and enhancing the long-term prognosis of DFSP patients. In the context of DFSP, combining different treatment modalities that target multiple pathways involved in tumor growth and progression can have synergistic effects (Miller et al., 2005[129]; Park et al., 2018[141]). It has been demonstrated that combining surgery and radiation therapy improves local control rates and overall survival in DFSP patients (Li et al., 2020[110]). Radiation therapy can target residual tumor cells and micrometastases, reducing the risk of local recurrence, whereas surgery aims to remove the tumor mass. By integrating these modalities, clinicians can achieve a more comprehensive and effective approach to tumor control (Li et al., 2020[110]). Similarly, targeted therapy combinations can produce synergistic effects in DFSP (Nishida et al., 2011[136]). Targeting the PDGFR pathway, the tyrosine kinase inhibitor imatinib has exhibited remarkable activity against DFSP (Nishida et al., 2011[136]). However, not all DFSP tumors respond equally to imatinib, and over time, resistance can develop. Combining imatinib with other agents, such as methotrexate or chemotherapy drugs, may overcome resistance and improve treatment outcomes (Wunder et al., 2007[205]). These combination strategies can act on various molecular targets or pathways, thereby increasing the likelihood of achieving a durable response and overcoming drug resistance.

In order to effectively address resistance in DFSP, it is imperative to thoroughly comprehend the underlying mechanisms that contribute to treatment resistance (Chen et al., 2022[33]). Resistance in DFSP can arise from various genetic and molecular changes, such as PDGFRB gene mutations and alternative signaling pathways activation (Capdeville et al., 2002[30]). Through the identification of these mechanisms of resistance, researchers can formulate targeted therapeutic approaches or combination strategies to counteract them effectively. An illustration of this can be seen in the advancement of second-generation tyrosine kinase inhibitors and the utilization of a combination of multiple targeted agents with distinct mechanisms of action. These approaches have the potential to effectively address resistance and improve treatment outcomes in the context of DFSP (Ghione et al., 2020[63]). In addition, current research focuses on identifying biomarkers or predictive factors that can guide treatment selection and enhance patient outcomes (Cheng et al., 2021[36]). These biomarkers can assist in identifying patients who are more likely to respond to particular therapies or combinations, allowing for a more individualized and tailored treatment approach (Cheng et al., 2021[36]). In addition, developments in genomic profiling and molecular characterization of DFSP tumors may help identify novel therapeutic targets and pathways, enhancing treatment efficacy and overcoming resistance.

## Future Perspectives and Challenges

The perspectives and challenges in DFSP research associated with potential prognostic markers and predictive biomarkers are essential provinces of research that hold great promise in advancing the management of this rare soft tissue sarcoma (Blay et al., 2020[19]). Prognostic markers predict the course of the disease, whereas predictive biomarkers identify patients who are more likely to respond to specific therapies (Nicolini et al., 2018[135]). In this section, the prospects and challenges associated with the identification and use of these markers in DFSP will be discussed. One promising future direction is identifying and validating novel prognostic markers for DFSP (Huang et al., 2022[80]). Currently, tumor size, location, and invasion depth are crucial prognostic factors in DFSP (Bowne et al., 2000[23]). These factors, however, do not fully capture the disease's heterogeneity and variable clinical behavior. As a result, additional molecular markers or genetic alterations that can predict disease progression, recurrence, and overall survival are required. Comprehensive genomic profiling, transcriptomic analyses, and other high-throughput techniques may be used to identify specific alterations or gene expression patterns linked to aggressive disease demeanor.

Furthermore, predictive biomarker development is critical for guiding treatment decisions and optimizing therapy in DFSP (Twomey et al., 2017[182]). Currently, identifying patients who are likely to respond to targeted therapies such as imatinib is difficult. While specific genetic changes, such as PDGFB gene rearrangements, are linked to imatinib sensitivity, there is still significant treatment response heterogeneity within this subgroup (Mertens et al., 2016[128]). To guide treatment selection and personalize therapy for DFSP patients, future research should aim to identify additional predictive biomarkers, such as changes in downstream signaling pathways or markers of drug sensitivity or resistance. The disease's rarity is one of the challenges in identifying and utilizing prognostic and predictive markers in DFSP (Beck et al., 2010[15]). Because DFSP accounts for a small proportion of soft tissue sarcomas, large cohorts of patients for robust analyses are difficult to obtain. Collaborative efforts, data sharing, and multicentered studies are critical to overcome this challenge and ensure an adequate sample size for meaningful analyses (Mahony et al., 2006[121]). Furthermore, establishing international registries and biobanks dedicated to DFSP can facilitate the collection of clinical and molecular data, thereby accelerating research progress in this area.

Another challenge is the molecular and histological heterogeneity of DFSP (Hao et al., 2020[70]). DFSP has several subtypes, including fibrosarcomatous and myxoid variants, each with distinct molecular and clinical characteristics. This heterogeneity should be considered when identifying prognostic and predictive markers and subtype-specific markers (Xing et al., 2023[206]). Integrating molecular and histopathological data can improve prognosis and prediction accuracy, but standardized approaches and guidelines are required to ensure consistency across different centers. Finally, translating prognostic and predictive markers into clinical practice is critical. To demonstrate their utility in guiding treatment decisions and improving patient outcomes, biomarkers must be validated in independent cohorts and subjected to rigorous clinical trials (Dobbin et al., 2016[48]), summarized in Table 3(Tab. 3) (References in Table 3: Delyon et al., 2021[45]; Durack et al., 2021[50]; Eilers et al., 2015[51]; Fiore et al., 2005[54]; Greco et al., 2001[67]; Kamar et al., 2013[90]; Sjöblom et al., 2001[166]; Wang et al., 2015[192]; Yuan et al., 2021[214]). In addition, companion diagnostic tests capable of detecting these markers in routine clinical practice are required for widespread adoption. Overcoming difficulties associated with the rarity and heterogeneity of DFSP and translating markers into clinical approaches is critical for their successful implementation. Collaboration, standardization, and multicentered studies are critical to furthering our understanding of DFSP and improving patient care through prognostic and predictive markers (Linch et al., 2014[111]; Jain, 2005[83]).

The future perspectives and challenges associated with advances in personalized medicine for DFSP have the potential to significantly improve patient outcomes, but they must be carefully considered (Avramescu et al., 2021[9]). The goal of personalized medicine is to tailor treatment plans to specific patient characteristics, such as the molecular profile of the patient's tumor. While significant progress has been made in this field for DFSP, several future perspectives and challenges must be addressed (Wang et al., 2023[193]). The identification of additional molecular alterations and biomarkers associated with DFSP represents one perspective for the future. Additional genetic mutations, alterations in gene expression patterns, or modifications in epigenetic mechanisms may potentially contribute to the pathogenesis and advancement of DFSP (Mashima and Sawada, 2021[122]). The utilization of comprehensive genomic profiling and transcriptomic analyses can be instrumental in the identification of these alterations, thereby facilitating a more profound comprehension of the disease's underlying biology. The integration of multi-omics data, such as genomics, transcriptomics, and proteomics, can provide a more complete view of DFSP and guide the development of personalized treatment strategies (Nassar et al., 2020[132]).

The advancement of functional characterization of identified molecular alterations is an additional outlook for the future (Lee et al., 2023[104]). Understanding the functional consequences of genetic alterations and their effect on the biology of tumors can provide insight into potential therapeutic targets. Preclinical studies utilizing patient-derived models, such as PDX or organoids, can assist in elucidating the mechanisms by which specific alterations contribute to tumor growth and in identifying novel vulnerabilities (Sampson et al., 2013[155]). The integration of functional investigations and molecular profiling has the potential to enhance the advancement of targeted therapeutic interventions and facilitate the customization of treatment strategies for individual patients (Harris et al., 2016[71]). Moreover, the integration of artificial intelligence (AI) and machine learning algorithms holds promise for enhancing the precision and effectiveness of DFSP's personalized medicine (Peleva et al., 2023[142]). Large datasets, such as clinical, genomic, and imaging data, can be analyzed by AI to identify patterns, predict treatment responses, and guide treatment decisions. By leveraging AI algorithms, clinicians have access to comprehensive and real-time data, allowing for more precise and individualized treatment strategies (Yoon et al., 2022[211]). However, data quality, standardization, and the interpretability of AI models must be addressed to ensure their ethical and dependable application in clinical practice.

Despite these future prospects, DFSP still faces obstacles in the advancement of personalized medicine (Balmaña et al., 2011[13]). The disease's rarity makes obtaining sufficient patient samples and constructing large-scale datasets challenging. Collaborative efforts, data sharing, and the establishment of international registries can aid in addressing this challenge and facilitate the collection of data required for effective personalized medicine approaches (Khleif et al., 2010[94]). Furthermore, it is imperative to validate the identified biomarkers and therapeutic targets through prospective clinical trials in order to guarantee their clinical applicability and facilitate the translation of research discoveries into enhanced patient care. The cost and accessibility of advanced technologies, such as comprehensive genomic profiling and AI algorithms, are another obstacle (Mateo et al., 2022[123]). It is possible that these technologies are not widely accessible or affordable in all healthcare settings, limiting their widespread adoption. Efforts should be made to increase accessibility, reduce costs, and establish guidelines and standards for the routine clinical integration of personalized medicine approaches (Golubnitschaja et al., 2012[65]).

## Conclusion

The DFSP is a rare cancer with unique molecular characteristics. It is crucial to comprehend the molecular aspects of DFSP to enhance diagnosis, prognosis, and treatment methods. Various techniques such as FISH, CMA, PCR, RNA-seq, and NGS have provided valuable insights into the genetic alterations and gene expression patterns associated with DFSP. Gene expression profiling in DFSP has identified specific molecular signatures, indicating the disease's heterogeneity and potential therapeutic targets. The PDGFR and FGFR signaling pathways play significant roles in the development and progression of DFSP. Targeting these pathways as a treatment for DFSP has shown promise. Surgical resection is the primary treatment for DFSP, but emerging therapeutic strategies such as immunotherapies, immunomodulatory approaches, and immune checkpoint inhibitors are being investigated as potential options. Combination approaches such as rational combination therapies aim to exploit synergistic effects and overcome resistance in DFSP management.

## Declaration

### CRediT authorship contribution statement

Conceptualization - HS, HBC, DSM, and TV; Writing-original draft - HS, HBC, and SM; Writing-review & editing - HS, MSM, SM, MGA, BKS, AKM, AK and HC; Supervision - AKM, AM, MGA and HC; Formal analysis -HS, SM, MGA, and AK; Visualization - SM.

### Acknowledgments

Harpreet Singh would like to acknowledge School of Pharmaceutical Sciences, IFTM University, India for providing facilities to perform research-oriented works and drafted this manuscript. Sourav Mohanto and Mohammed Gulzar Ahmed would like to acknowledge Yenepoya (Deemed to be University) for continuous support on various research related activities. The figures were drawn via Biorender.com.

### Funding

Not applicable.

### Institutional review board statement

Not applicable.

### Informed consent statement

Not applicable.

### Data availability statement

Not applicable.

### Declaration of interest

The authors declare that they have no known competing financial interests or personal relationships that could have appeared to influence the work reported in this paper.

## Figures and Tables

**Table 1:**
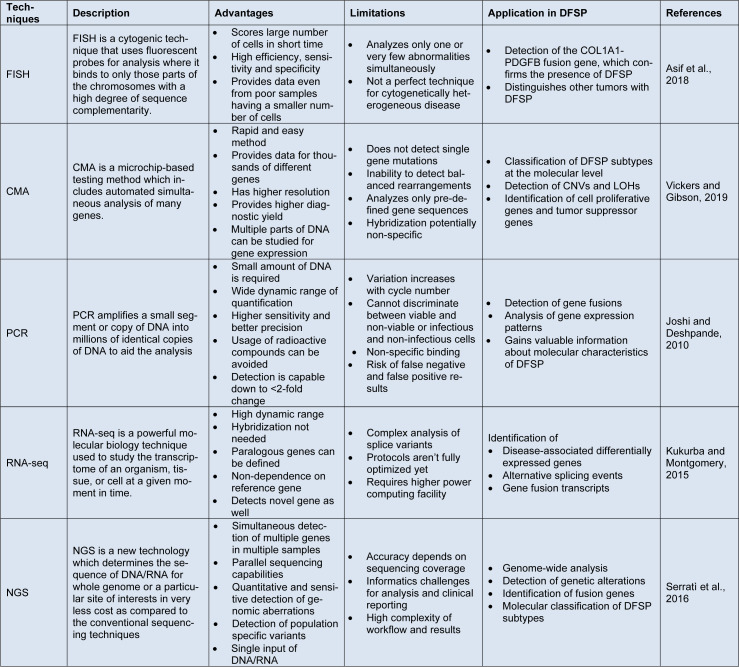
Summarization of various genomic profiling techniques, their characteristics, and limitations for DFSP genetic landscape

**Table 2:**
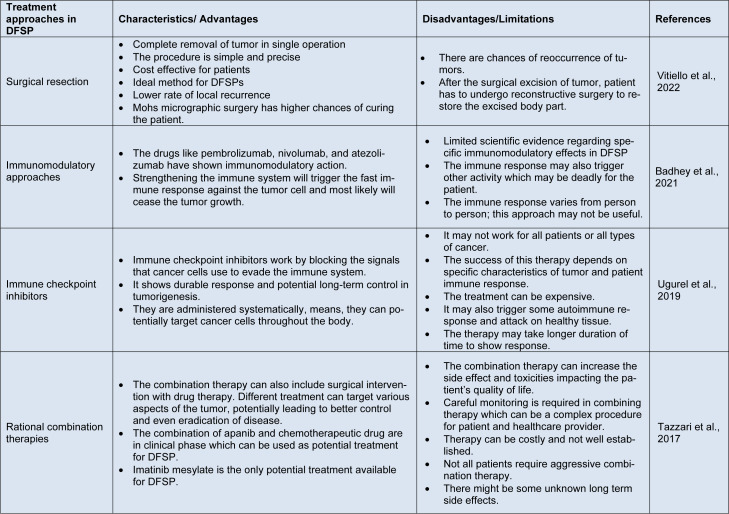
Summarization of various therapeutic/ treatment strategies and their advantages, and disadvantages in DFSP management

**Table 3:**
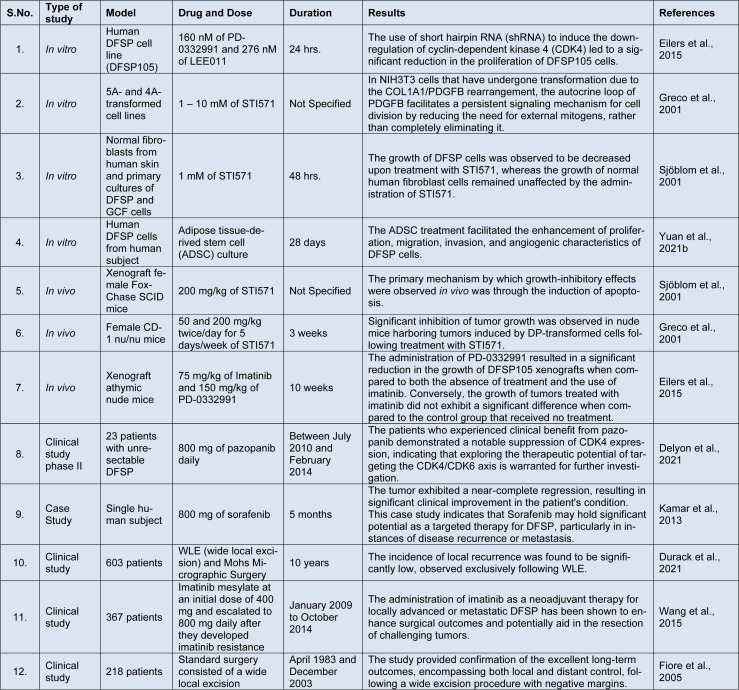
Various recent investigations on DFSP management and treatment via conventional and combinatorial therapeutics

**Figure 1:**
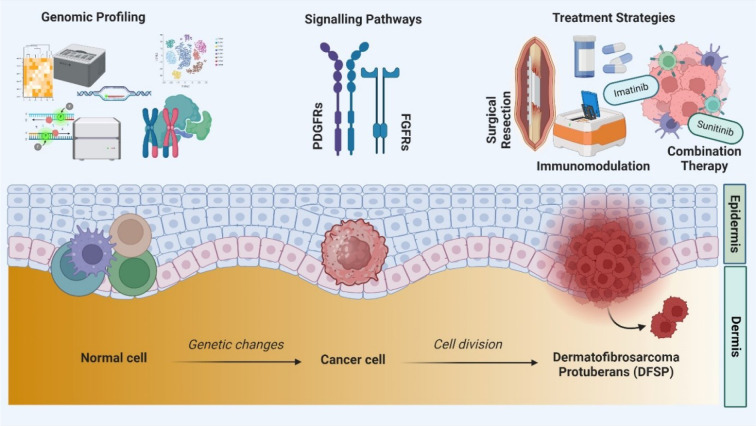
Graphical abstract
